# Targeted delivery of gold nanoparticles by neural stem cells to glioblastoma for enhanced radiation therapy: a review

**DOI:** 10.3934/Neuroscience.2022017

**Published:** 2022-07-08

**Authors:** Mogesh Sababathy, Ghayathri Ramanathan, Suat Cheng Tan

**Affiliations:** 1 Department of Veterinary Pathology and Microbiology, Faculty of Veterinary Medicine, Universiti Putra Malaysia, Serdang, Malaysia; 2 Institute of Biological Sciences, Faculty of Science, University of Malaya, Kuala Lumpur, Malaysia; 3 School of Health Sciences, Health Campus, Universiti Sains Malaysia, 16150 Kubang Kerian, Kelantan, Malaysia

**Keywords:** glioblastoma, gold nanoparticle, radiosensitization, neural stem cells, targeted delivery

## Abstract

Glioblastoma (GB) is the most malignant subtype of brain cancer derived from astrocytes in the brain. Radiotherapy is one of the standard treatments for GB patients, but its effectiveness is often limited by the radioresistance of aggressive GB cells. Higher dose of radiation needs to be applied to GB patients to eliminate these stubborn cells, but this also means more side effects on the adjacent healthy cells because the radiation beam could indistinguishably harm all cells exposed to it. In order to address this problem, various strategies have been studied to enhance the radiosensitivity among the radioresistant cell populations for targeted eradication of GB without harming other surrounding healthy cells. One of the promising strategies for radiosensitization is to use gold nanoparticles (AuNPs) which can enhance photoelectric effects within the radioresistant cells for higher killing efficiency even at low doses of radiation. Nonetheless, there is no evidence showing the capability of these nanoparticles to travel to brain tumor cells, therefore, the application of this nanotechnology is very much dependent on the development of a suitable carrier to deliver the AuNPs to the GB tumor sites specifically. In this review article, we discussed the potentials of neural stem cells (NSCs) as biological carriers to carry AuNPs to targeted GB tumor sites and provided new insights into the potential of NSC-based targeted delivery system for GB treatment. The information reported here may pave a new direction for clinical transformation of next-generation nanoparticle-assisted radiotherapy to optimize the efficacy of radiotherapy for GB treatment.

## 1. Introduction

### 1.1. Glioblastoma (GB)

Glioblastoma (GB), or formerly known as glioblastoma multiforme (GBM), is grade IV diffuse astrocytic tumor known to be the most malignant and most invasive type of primary brain tumor. It is characterized by its unregulated proliferation, biological heterogeneity, ability to metastasize and hallmark histological features such as hypercellularity, nuclear atypia, microvascular proliferation and necrosis [1]. The tumor is very malignant due to its highly invasive nature as well as the support from its huge network of blood supplies due to rapid angiogenesis rate [2]. Since they can grow very aggressively, the clinical symptoms of GB are generally related to the pressure increase inside the brain. Some common manifestations include persistent headache, nausea, and vomiting. Additional symptoms also may develop in relation to the location of the tumor in the brain such as blurred vision, speech and balance difficulties, memory loss, confusion or a decline in brain function and even personality changes [3].

GB makes up 80% of malignant brain tumors that originate from the star-shaped astrocytic glial cells and accounts for the rising incidence of brain cancers each year globally [4]. Moreover, this disease also has the lowest median survival rate among all types of tumor occurring in the central nervous system (CNS). In general, the median survival of GB patients from initial diagnosis is less than 15 months, with a 2-year survival rate as low as 26–33% [5]. Despite the growing understanding in the biology of cancer and advancements in medical technology, recent studies still disappointingly showed no sign of improvement in the survival rates among GB patients. The poor prognosis rate of GB is mainly due to the localization of the tumor, highly heterogeneous nature of the disease, resistance to conventional therapies, high recurrence rate as well as the limited capacity for the brain to self-repair [6]. Moreover, there is also evidence showing that extracellular vesicles secreted by glioma stem cells are involved in the glioma resistance and progression [7].

Albeit extensive research has been done over the last few decades in the efforts to enhance our understanding about the causality of GB and its underlying mechanisms, a great unknown still remains. Several risk factors of GB have been laid out by researchers and have been widely accepted in the medical arena, including the unhealthy lifestyle, prolonged exposure to ionizing radiations and carcinogens, accumulation of molecular alterations as well as inheritance or family history. However, these risk factors are either accountable to only a very limited type of brain malignancies or their actual effects are still highly uncertain [3].

### 1.2. Current GB treatments and the challenges

Similar to most cancer types, standard therapies for GB consist of maximal surgical resection, followed by radiotherapy plus concomitant and maintenance with chemotherapy [3]. Usually, a total resection of tumor tissue via surgery is the first-line treatment given to the patients diagnosed with GB. Ideally, it would yield a positive outcome for the patients if the surgical resection could successfully remove total tumor tissue before it spreads into the interstitial brain areas. However due to its invasive behavior, total removal of metastatic GB brain tissue can be challenging even for a skilled surgeon [3]. Moreover, the tumors are often located at the remote areas of the brain, causing the surgical removal of the total tumor a highly risky procedure. On top of that, it would expose patients to risks of infection, bleeding, and nerve damage after surgery, which may cause interruption in speech, motor functions and sensory perception of the patients [8]. Therefore, in most cases, microscopic tumor foci left mislaid after surgery and this led to recurrence of the cancer.

To date, there is no established standard of care for recurrent GB after first surgery. Available options for patients include second surgery, re-irradiation, and combined modality therapies [9],[10]. In a clinical study reported by Montemurro et al., 63 patients with recurrent GB were offered second surgery as part of the treatments between year 2006 and 2020 [11]. The results reported that patients with an initial gross-total resection (GTR) had a maximized overall survival (OS) after a second GTR at recurrence. However, another study reported by Sacko et al. stated that repeated surgeries were associated with temporary or permanent postoperative morbidity in a substantial number of cases [12]. In addition, there is also no consensus in the literature on the role of reoperation in the management of the GB patients and the evidence-based prediction on which GB patient is most likely to benefit from the second surgery [13]. Therefore, the role of reoperation for recurrent or progressive disease still remains controversial [14]. Given the etiological complexity and the aggressive nature of GB, effective surgical treatment options for GB are still a major unmet medical need to date.

Thus, patients with advanced stages of GB may have to undergo intense treatment schedules of radiotherapy and chemotherapy following surgeries. The current standard clinical chemotherapy drug for GB is Temozolomide (TMZ), which is generally administered every day during radiation therapy followed by six cycles during the maintenance phase [15]. TMZ is a DNA alkylating agent that preferentially methylates DNA at the N7 position of guanine, O3 position of adenine and O6 position of guanine. Alkylation of the O6 site on guanine leads to the formation of O6-methylguanine adducts and results in the insertion of thymine residues instead of cytosine. The mutations induce the formation of single- and double-stranded DNA breaks, resulting in cell cycle arrest at G2/M and apoptosis, which eventually lead to cancer cell growth inhibition [16]. Concomitant therapy using both TMZ and radiation improved overall survival of newly diagnosed adult GB patients compared to those treated with radiation alone (14.6 vs. 12.1 months median survival) [17]. Nonetheless, acquired chemoresistance to TMZ developed in cancer cells has been shown to be a major limitation to this therapy. It was found that more than 90% of recurrent gliomas show no response to the second cycle of chemotherapy [16]. Many studies have proven that the development of GB resistance to TMZ is driven primarily by a unique population of undifferentiated and highly tumorigenic cancer stem cells known as glioma stem cells [18],[19]. One of the major mechanisms of TMZ resistance induced by these glioma stem cells is due to the enhanced activity of a DNA repair enzyme known as O6-methylguanine-DNA-methyltransferase (MGMT) in the cells. MGMT repairs TMZ-induced DNA lesions by removing the alkyl groups from the O6 position of guanine directly, which counteracts the TMZ-induced DNA alkylation of the O6 site on guanine [20]. Therefore, high expression of MGMT is very often linked to TMZ resistance and a bad prognosis of patient survival.

On the other hand, radiation therapy is a highly effective tool for cancer treatment. Together with surgery and chemotherapy, they make the first line therapeutic component of cancer management, conferring survival and palliative benefits. The effectiveness of radiation depends on the linear energy transfer, total dose, and radiosensitivity of the targeted cells or tissues. Besides, the extent of resection during first surgery and the expression of chemical markers such as Ki-67 could significantly provide predictive values for the prognostic effects of radiation [21]. Ki-67 is a nuclear protein present during all active phases in a cell cycle (G1, S, G2, and mitosis), but absent in quiescent cells (G0). The Ki-67 proliferation index is one of the immunohistochemical markers for the evaluation of intracranial tumor proliferation. It is a useful indicator to differentiate between high and low-grade gliomas, in which low Ki-67 expression levels are directly associated with grade II–III whilst high Ki-67 expression levels are directly associated with high grade IV gliomas [21]. Understanding the chemical marker expression during or after the course of irradiation will lead to improvements in therapeutic efficacy and potentially, benefitting a significant proportion of cancer patients. However, despite the fact that Ki-67 is strongly associated with tumor cell proliferation and growth, its expression level alone cannot alter the survival in GB patients [22].

Moreover, chemo-radiation treatments also are known to cause unwanted side effects on patients such as fatigue, headaches, nausea, vomiting, hair loss, and weakness. These side effects had been widely evident to affect the patients' mental health, resulting in patients experiencing various levels of psychological distress, anxiety, depression, and even hallucination [23]. As a consequence, the therapies may progressively lead to a decline of the patients' overall health and reduce their compliance to the treatment, causing the survival rate to deteriorate greatly. Thus, an alternative treatment targeting GB specifically to reduce unwanted side effects and increase patients' quality life is necessary.

### 1.3. Enhanced radiosensitivity of GB cells for optimized radiation therapy

Radiation therapy is one of the most common treatments for cancers especially for tumor tissues that are not easily accessible or too risky for surgical removal. The idea of using radiation in cancer treatment emerged in 1895 when Wilhelm Conrad Roentgen, a Nobel Prize winner in Physics, discovered the X-ray. It did not take long after that, X-rays were used in cancer treatments by the E. H. Grubb company at the suggestion of Doctor Ludlam [24]. Radiotherapy works by emitting high-energy waves to kill or shrink tumor cells by targeting their DNA which may take days or weeks of continuous treatment for the cells to completely die and eradicate from the body [25].

According to the American Cancer Society [26], conformal radiation therapy, conformal proton beam radiation therapy, stereotactic radiosurgery, and intraoperative radiation therapy are several variations in current radiation therapy for cancer treatment. The advancement of radiotherapy is driven by the call to search for targeted radiotherapies that are not only able to precisely locate and destroy the tumor in patients but also able to protect surrounding healthy tissues from getting exposed to the high dose of radiation. Currently, in the clinical setting of GB treatment, patients are given radiation therapy combined with radiosensitizer drugs such as Temozolomide [27] that help to increase the vulnerability of tumor cells to radiation. However, multiple cases have reported that these drugs show aftermath effects on the patients such as changes in the skin, exhaustion, hormonal changes and may even lead to secondary cancers in worse cases [28]. Therefore, there is a pressing need for novel and innovative treatment strategies to enhance radiosensitivity of GB cells for optimized radiation therapy.

Scientists around the world over the last decade, have been looking into exploring the potentials of integrating nanotechnology with cancer treatment especially to enhance radiosensitivity of cancer cells in order to overcome the resistance to radiation and reduce harmful effects of ionizing radiation to normal cells. Among them, gold nanoparticles (AuNP) have emerged as a novel alternative method that is highly efficient, relatively less laborious, inexpensive, and most importantly able to exert significant effects as a radiosensitizer.

In this review article, unique characteristics of AuNP that make it a good radiosensitizer in cancer radiotherapy were discussed. We also discussed the targeted delivery of AuNP to GB sites using neural stem cells (NSC) to provide a theoretical basis for the future development of targeted AuNP delivery systems. The literature review reported here also may provide new directions for the optimization and clinical transformation of next-generation nanoparticle-assisted radiotherapy to increase the efficacy of radiotherapy and prognosis rate among GB patients.

## 2. Gold nanoparticles (AuNPs)

AuNPs have a long history in chemistry. It was as early as in the fifth century BC where researchers from different parts of the world discovered astonishing properties of gold in medical application. This includes the utilization of gold wire in jaw fracture, to heal fistulas, and hemorrhoids [29],[30]. The contemporary era of AuNP synthesis was initiated by Michael Faraday about 150 years ago [31]. He was the pioneer in identifying the distinct characteristics of colloidal gold solutions which vary from the bulk gold and made a big step forward in nanoparticle research. Nonetheless, most studies devoted to the application of gold nanomaterial in medicine only started to make remarkable breakthroughs after many decades due to the limitations of technology back then.

Over the years, there has been an immense growth of interest in the utilization of nanotechnology in a wide range of biological areas that has progressively led to various novel findings including in the application of gold nanomaterial for numerous medical treatments. Credits to decades of extensive research on fundamental understanding around potential uses of AuNPs in medicine, AuNPs have been functionalized using various methods, and they can be used to detect, visualize, and recognize various biological signatures, to enhance efficiency of drug delivery systems or to treat deadly diseases like cancer using treatment methods such as selective photothermal therapy (PTT), photodynamic therapy (PDT), and radiation therapy [32].

### 2.1. Synthesis of AuNP

Studies have shown AuNPs developed in a wide range of sizes and structural variations such as gold nanorods, gold nanostars, gold nanorings, gold nanocages, and hollow gold nanoshells ranging from 1 to 100 nm [33]. AuNP is highly malleable and easily producible with two classic methods known as the Turkevich method and the Brust-Schiffrin two-phase synthesis method.

The Turkevich method utilizes citrate reduction of hydrogen tetrachloroaurate (HAuCl_4_) to produce gold nanospheres [30]. The Turkevich method was further advanced by Frens who altered the gold-to-citrate ratio to control particle size [34]. Spherical AuNPs with diameters between 10 to 20 nm and even larger (~100 nm) in diluted forms were produced using this modified method. However, these citrate-stabilized AuNPs can undergo irreversible aggregation during the functionalization process with thiolate ligands.

On the other hand, Brust-Schiffrin two-phase synthesis method utilizes sodium borohydride (NaBH_4_) as a reducing agent and a phase transfer reagent known as tetraoctylammonium bromide (TOAB) to create organic soluble alkanethiol-stabilized AuNPs [35]. This method is popularly known for its higher stability and lower dispersion when compared to most other AuNPs. Its high stability is mainly due to the synergic effect of the strong thiol and gold interactions and the van der Waals attractions between the neighboring ligands. These nanoparticles can be thoroughly dried and redispersed in solution without any aggregation, making them excellent precursors for further functionalization [36].

### 2.2. Unique characteristic of AuNP

A wealth of studies can be seen done over the last century to evaluate the efficiency of AuNP production. This has led to producing multiformed AuNPs with unique chemical and physical properties which are beneficial to humankind especially in biological and medical research [33]. For example, the photoacoustic and photothermal characteristics enable AuNPs to utilize light at certain wavelengths which is well-established as localized plasmon surface resonance (LPSR) [37]. This strengthens the tumor hyperthermia treatment procedures and medical imaging techniques. Besides, the size and shape of AuNPs can be altered to influence the photoacoustic and photothermal characteristics. Hence, it permits the AuNPs to absorb near-infrared (NIR) light in the wavelength range up to 1,000 nm which is sufficient for long tissue penetration depth, making it an ideal tool for in vivo deep tissue imaging and therapy [38]. Furthermore, enzymes produced in human bodies cannot break down the nanosize AuNPs, permitting them to be transported into the kidney and excreted out from the body via the urinary system as waste products [39].

### 2.3. Safety and Toxicity of AuNP

There has been a tremendous evolution in the AuNP applications over the years. However, there are some crucial concerns prior to the application of this technology in the clinical settings. Currently, the introduction of AuNPs into clinical practice is restricted, with only a few clinical trials approved by the FDA for cancer treatment [40]. Safety issues such as the toxicity level of AuNPs and its biocompatibility have significantly impeded the progress in nanotechnology developments [41]. Although AuNPs generally are non-reactive at room temperature, resistant to corrosion, and stable at high temperature (high melting point) [42], it has been proved that AuNP toxicity can be influenced by the alteration of AuNP composition. For example, the combination of AuNPs with methotrexate (MTX) has been reported to induce cytotoxicity in several cancer cell lines [43].

The toxicity of AuNPs has been widely described in numerous publications [41],[44], however some still reported otherwise [45]. Such inconsistency in the results is due to the varied physicochemical characters of AuNPs used in different studies such as the sizes, shapes, surface chemistry, stabilization coatings, as well as the administration methods (dosage, duration, route of administration, etc.) which may incite to different toxic effects of the AuNPs [46]. Therefore, it is very important to evaluate the toxicity of AuNPs prior to in vivo or clinical studies. The assessment of AuNPs could be done based on the potential hazards of AuNPs which included the in vivo modification of AuNPs, the interaction and catalytic properties of AuNPS in biological system and the distribution and accumulation of AuNP in the liver and other organs based on the Safety Assessment Guidelines approved by the Organisation for Economic Co-operation and Development (OECD) Guidelines for the Testing of Chemicals [47],[48]. A number of in vitro and in vivo studies have been conducted to evaluate the potential toxic effects of AuNPs in various cell lines such as human keratinocyte cells (HaCaT) [49], human colorectal adenocarcinoma cells (HT29) [50], and in animal models such as mice [47], rats [51], rabbit [52], zebrafish [53] and even the *Drosophila melanogaster* [54]. Despite the inconsistencies in reported AuNP toxicological data, it is generally found that the toxicity is size- and dose-dependent whereby the smaller sized and higher levels of AuNPs lead to higher cytotoxicity with unintended side effects on human health [46].

### 2.4. Potential of AuNP to enhance radiosensitivity

Radiosensitizers are chemical compounds that are able to enhance cell sensitivity to radiotherapy by altering the cell activity that modulate the lethal effects of radiation. Radiosensitizer-aided radiotherapy amplifies the effectiveness of tumor killing compared to the conventional radiotherapy method by enhancing the susceptibility of cancer cells towards the high energy photons from radiation therapy. The potential of high-Z metals such as gold, gadolinium, silver, and bismuth in enhancing radiosensitization effect was first observed in patients with metal implants who received radiotherapy for the treatment of mandibular [55],[56]. High-Z materials are material with high atomic number (Z) of protons in the nucleus. These materials can enhance radiosensitivity on cells based on two major mechanisms that are the physical dose enhancement and increased biological reactions in the tissue [56].

Physical dose enhancement by high-Z material is mainly observed via the photoelectric effect which occurs when the material interacts with the relatively low energy ionizing radiation (< 60 kiloelectronvolts, keV). The photoelectric effect results in the emission of Auger electrons which have a very high linear energy transfer that is highly toxic to the cells. The occurrence probability of the photoelectric effect increases proportionately with the number of atoms of the absorber, therefore, AuNP with high atomic number could significantly increase the local photoelectric interaction and energy deposition, typically 10 to 150 times more, at the target site. Enhanced physical dose at the target site subsequently led to increased biological reactions in the tissue that could induce cytotoxicity by the degradation of phospholipids, as well as disrupt the cell membrane permeability. Additionally, it also induces generation of ROS which accelerates DNA lesions and leads to cell death [57]. Therefore, taken together, by applying AuNP as a radiosensitizer on targeted cancer cells, radiologists can maintain a relatively lower dose of radiation power and thus, can reduce the side effects on surrounding non-target cells. Numerous in vitro and in vivo studies had been done to evaluate the efficacy of AuNPs as radiosensitizer [58],[59]. For example, a study by Anijdan et al. showed that local dose enhancement can be achieved by the AuNPs in melanoma tumor bearing mice [60]. Another study by Choi et al. also demonstrated an enhanced radiosensitizing qualities induced by AuNPs in a mouse tumor xenograft model of breast cancer [61]. To date, several clinical trials involving AuNPs are ongoing [59]. However, the translation of AuNPs into clinics is an expensive and time-consuming process that is associated with two main challenges: 1) requires extensive toxicity study (as mentioned in earlier section) and 2) complex delivery of AuNPs to targeted tumor sites.

### 2.5. Targeted Delivery of AuNP to Tumor Sites

Radiosensitization by AuNPs in cancer treatment is seen as a loophole still by many researchers since there is no substantial evidence that the AuNPs are able to home to cancer site specifically. Random delivery of AuNP via systemic route will deliver AuNP to the whole body randomly and thus increase the side effect of radiosensitization on non-targeted tissue. In order to address this problem, a specific delivery agent must be carefully opted. According to previous studies, the important characteristics of an ideal delivery agent in cancer therapy including; first, the carrier must possess tumor-selective migratory capacity; second, the carrier must be able to uptake the therapeutic agent and protect it from the host immune system and third, the agent must not have any significant chemical/biological interaction with the therapeutic agent which could interfere the therapeutic potential/function [62],[63].

To date, various carrier-mediated transports have been considered as vehicles to be used for delivery of anticancer agents such as lipid-bilayer vesicles (liposomes) [64], polymeric micelles [65], and modified mammalian cells [66]. Even though many of these agents showed promising potential for targeted delivery, most were unsuccessful to reproduce such effects in brain disease, when systemically administered in vivo. The major reason of the limited success is the incapability of the agents to cross the blood–brain barrier (BBB) and to penetrate into the brain tumor tissue [67].

Interestingly, as a solution to mitigate these challenges, researchers have suggested an innovative delivery system by using stem cells as a carrier vehicle to transport AuNP across the BBB to the tumor areas due to their unique ability to cross BBB and home to brain tumor sites.

## 3. Neural Stem Cells (NSCs)

### 3.1. Isolation and characterization of NSCs

NSCs are a large group of undifferentiated ectodermal progenitor cells located within the subventricular zone of the lateral ventricular, the subgranular zone of the hippocampal dentate gyrus, and external germinal layer of the cerebellum. NSCs possess self-renewable properties and express multipotency to differentiate into several subtypes of brain cells namely neurons, astrocytes, and oligodendrocytes [68]. For almost a century, scientists have believed that primate brains do not regenerate or add new neurons after maturity. However, controversies arise when NSCs were first identified within the CNS in 1992 [69]. The presence of NSCs challenged the generally accepted paradigm that the brain is a postmitotic organ and provides a potential alternative therapeutic tool to regenerate damaged brain cells after stroke.

Isolation of NSCs from their niche is a crucial step to unravel mechanisms underlying the cellular and molecular activities of the brain cells, thus, allowing for better understanding of neurological diseases' pathophysiology and etiology related to this cell type. NSCs can be isolated and expanded in vitro based on the identification of specific cell markers such as Nestin, Vimentin, Sox 2, and CD133 [70],[71] using neurosphere culturing technique [72]. This isolation technique is able to maintain NSC self-renewal and multipotency in monolayer culture and it is relatively easier, faster, highly reproducible, and is able to isolate a sufficient number of cells from only one animal.

### 3.2. Therapeutic potentials of NSC

Since their identification, NSCs have been revealed to be a useful tool for a broad spectrum of applications, both in vitro as a model of neural development/survival study and in vivo as a source of cells for clinical studies for cell therapy in many neurodegenerative diseases [73],[74]. NSCs generally play a vital function in neural regeneration, regulate the brain homeostasis, and maintain its integrity throughout one's life. NSCs have exceptional potential to self-regenerate and repair damaged nervous systems. The regenerative properties of NSCs enable amelioration of brain injuries. They recover the damaged neural network by regenerating glial cells and neurons. A current finding illustrated the ability of NSCs to perform neurogenesis in the damaged human brain [75]. NSCs were also reported to play significant roles in managing the inflammation that occurs due to brain injury while activating neuroprotective activities.

### 3.3. Homing properties of NSCs

Recently, researchers reported another important characteristic of NSCs which is their unique tropism capacity to glioma sites [76],[77]. A tropism is a biological phenomenon in which an organism grows or turns its growing movement in response to an environmental stimulus. This growth or movement normally depends on the direction of the source of stimulus originated from a specific cell type of which the particular organism has evolved to preferentially target to or bind to. Tropism behavior is a multiplexed process which involves many steps. Most cells utilize these tumor-trophic properties to travel from origin position to a tumor at a distant area [78].

Tropism of NSCs to glioma sites has been first demonstrated by Aboody et al. in which they found that the transplanted stem cells gathered around metastatic tumor beds which were literally far from their site of original transplantation [79]. In the past years, various preclinical trials were advanced to explore the intrinsic glioma-tropic properties of NSC to make it a carrier of AuNP for targeted GB treatment. For example, Mooney et al. demonstrated an enhanced distribution of nanoparticles to tumor tissues by loading gold nanorods onto NSC carriers which showed a wider spread around the tumor site [80]. Although the specific mechanisms of tumor-homing properties are still unrevealed, it is hypothesized that the microenvironment of GB sites contribute significantly to the migration capacity of NSCs. An emerging tumor microenvironment is a complex and continuously evolving entity composed of tumor cells and tumor stromal cells such as stromal fibroblasts, endothelial cells, and immune cells like microglia, macrophages, lymphocytes, and the non-cellular components such as collagen, fibronectin, hyaluronan, and laminin which make up the extracellular matrix (ECM) of tumor microenvironment [81].

Tumor microenvironment plays essential roles in regulating initiation, progression, angiogenesis, and metastasis of cancer stem cells by creating a niche to maintain and nurture the cells [82]. There is growing evidence that shows multipotent stem cells are recruited into tumor microenvironment in response to multiple signals produced by cancer cells [83],[84]. There are many signals involved in the cross-talk between tumor-stem cells and other components of the tumor microenvironment. Several chemokines, cytokines, and growth factors are produced by tumor cells to recruit NSCs. A study by Xu et al. [85] found that the stromal cell-derived factor 1 (SDF-1)/C-X-C chemokine receptor type 4 (CXCR4) signaling pathway are among few of the many factors that were expressed during NSC migration. A similar finding was also pointed out by Heese et al. who focused on the role of scatter factor/hepatocyte growth factor (SF/HGF) in stimulating migration capacity of human NSCs [86]. In addition, a tumor-derived mediator known as vascular endothelial growth factor (VEGF) also was found to induce attraction of NSCs from distant sites in the adult brain [87].

**Figure 1.**
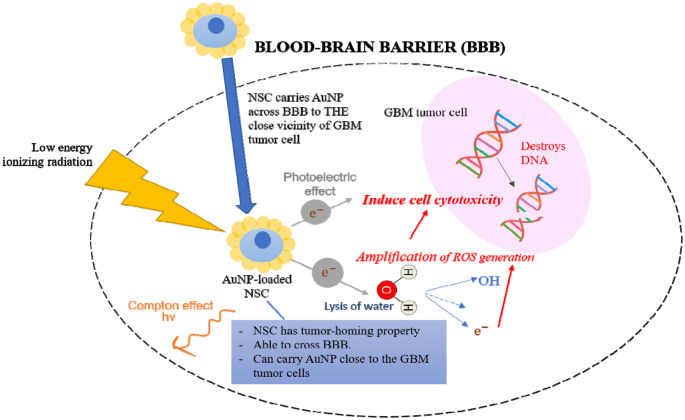
Illustration of radiosensitization by gold nanoparticle (AuNP)-loaded neural stem cells (NSC) on glioblastoma (GB) tumor cells. The NSC has tumor homing property which plays a crucial role in carrying the AuNP across the blood-brain barrier (BBB) to close vicinity to the targeted GB cell. Photoelectric effect is enhanced in the presence of AuNP when material interacts with the relatively low energy ionizing radiation. Besides, ionization of water molecules also releases free radicals and triggers amplified ROS generation which could induce cytotoxicity in the tumor cell. Taken together, these trigger DNA damage and destroy the tumor cells.

In summary, this article presented the potential of NSC in targeted delivery of AuNPs to glioma cells. The high atomic number gold nanomaterial could significantly increase the local photoelectric interaction and subsequently lead to increased cytotoxicity, generation of ROS, and DNA lesions as summarized in Figure 1. Nonetheless, substantial works need to be done in understanding the exact mechanism and safety profile of NSCs as carriers of therapeutic agents [88].

## 4. Conclusion and recommendations

This review article summarized the unique characteristics of AuNPs and their potential in enhancing radiosensitivity for cancer radiotherapy. Applications of AuNPs as radiosensitizers on target cancer cells could preferentially sensitize tumors to radiation whilst minimizing effects in normal tissues. Despite the potential of AuNPs to induce radiosensitization in cancer cells, there are numerous challenges towards the clinical translation. Among them, targeted delivery of AuNPs to tumor sites is the major issue. We believe that the application of stem cells as biological carriers may lead to breakthroughs as part of nanoparticle administration strategies for medical treatment especially for cancers. NSC-mediated delivery of AuNPs takes advantage of the innate capability of the stem cells to migrate and home to GB sites. They are capable of carrying AuNPs across the BBB and penetrate into the brain tumor tissue specifically. As mentioned before, future studies have to be focused on addressing the remaining challenges prior to making NSC a clinically viable option for AuNP delivery. Modalities to circumvent common challenges when designing a stem-cell-mediated targeted delivery system such as the stem cell collection and culture protocol standardization, the regulation of AuNPs uptake and the release mechanism by stem cells, the long-term health effects, and interactions of NSC with human immunological system must be tackled. It is also crucial to conduct future studies on calibrating AuNP dosage and physicochemical properties to steer clear of any possible toxicity effects, and elucidate underlying mechanisms and pathways involved within the tumor microenvironment that could affect the migration of AuNP-loaded NSCs to the GB sites. In conclusion, this review paper provided insights and future prospects of next-generation nanoparticle-assisted radiotherapy to increase the efficacy of radiotherapy and prognosis rate among GB patients.
